# The mechano-immunological barrier in fibrosis-associated lung cancer: targeting matrix stiffness and the Piezo1 axis for microenvironment normalization

**DOI:** 10.3389/fphar.2026.1845077

**Published:** 2026-08-07

**Authors:** Linfeng Guo, Jia Wang, Baoyi Ni, Jiakang Jiang

**Affiliations:** 1 Heilongjiang University of Chinese Medicine, Harbin, China; 2 Hongqi Hospital of Mudanjiang Medical University, Mudanjiang, China; 3 Mudanjiang Medical University, Mudanjiang, China; 4 The First Affiliated Hospital of Heilongjiang University of Chinese Medicine, Harbin, China

**Keywords:** extracellular matrix stiffness, mechano-immunology, mechanotransduction, non-small cell lung cancer, Piezo1

## Abstract

While mechanotransduction influences various lung cancer subtypes, this review focuses specifically on fibrosis-associated non-small cell lung cancer (NSCLC), where the progression from pulmonary fibrosis to malignancy is mediated by aberrant mechanotransduction. Progressive extracellular matrix (ECM) stiffening (8–25 kPa), mediated by lysyl oxidase (LOX)-dependent collagen crosslinking, establishes a “mechano-immunological barrier.” This barrier promotes malignant transformation and limits the efficacy of immunotherapy. Piezo1, a mechanically-activated cation channel, regulates this physical remodeling of the tumor microenvironment (TME). In the stiffened fibrotic stroma, mechanical tension activates Piezo1, causing intracellular Ca^2+^ influx. This influx bypasses canonical Hippo signaling to activate the RCC2-YAP axis and induces “epigenetic mechanical memory” via Rho/ROCK-mediated histone acetylation. Furthermore, Piezo1 modulates local immune responses. Mechanical stress activates Piezo1 in infiltrating T-cell, leading to epigenetic exhaustion and cytoskeletal impairment. Simultaneously, this mechanotransduction polarizes macrophages toward an immunosuppressive M2-like phenotype and mechanically induces PD-L1 expression on tumor cells. This review summarizes the Piezo1-mediated mechanotransduction networks involved in the fibrosis-to-cancer transition. We also discuss emerging “mechano-immunotherapeutics,” including targeted Piezo1 modulators, precision nanodelivery systems, and ECM-normalizing agents (e.g., LOX inhibitors and losartan). These strategies demonstrate synergistic effects when combined with immune checkpoint inhibitors. By softening the desmoplastic niche—a tumor-associated microenvironment characterized by excessive fibrous connective tissue and a dense ECM—and modulating mechanosensors, these interventions can convert immunosuppressive “cold” tumors into immunotherapy-responsive “hot” microenvironments.

## 1 Introduction

Idiopathic pulmonary fibrosis (IPF) and lung cancer share pathophysiological mechanisms, representing a biological continuum rather than independent comorbidities. Epidemiological evidence indicates that patients with interstitial lung diseases have a 2.16- to 2.91-fold increased risk of developing lung malignancies, with a lifetime prevalence of approximately 13.5% (Xu et al., 2025; Brown et al., 2019). These tumors predominantly develop within or adjacent to fibrotic and honeycombing lesions (Erten et al., 2026; King and Nathan, 2017). Histologically, they present primarily as squamous cell carcinoma and adenocarcinoma (Xu et al., 2025; Jafarinezhad and Yektakooshali, 2018). This spatial and clinical correlation suggests that the fibrotic pulmonary stroma functions as a pre-metastatic and pro-tumorigenic niche.

The transition from fibrosis to malignancy is driven by progressive ECM remodeling and aberrant mechanotransduction—the fundamental cellular process of converting physical mechanical stimuli into biochemical signals. In the fibrotic lung, the deposition of fibrillar collagens and LOX-mediated crosslinking increase tissue stiffness. This alteration increases the stiffness of normal lung parenchyma from 0.2–2 kPa to 8–25 kPa, a rigidity similar to that of solid tumors (Guo et al., 2022; Chang et al., 2015). Importantly, cellular responses vary across this stiffness gradient: tumor cells located in the stiffest regions (∼25 kPa, characteristic of the invasive front) exhibit pronounced EMT and maximal YAP activation Jiang et al., 2025), whereas cells in intermediate stiffness zones (∼8 kPa, typically within the hypoxic core) adapt primarily through metabolic reprogramming to maintain survival (Sohrabi et al., 2023). This biomechanical change acts as a physical driver of carcinogenesis. ECM rigidity interacts with cancer-associated fibroblasts (CAFs) and inflammatory networks, which are primarily regulated by TGF-β signaling. This interaction alters the metabolic and immunological microenvironment (Shi et al., 2020; Bhardwaj et al., 2025). Consequently, the fibrotic matrix forms a physical barrier that restricts cytotoxic T-cell infiltration and induces early PD-L1 upregulation. These changes establish an immunosuppressive environment prior to full malignant transformation (Park et al., 2024).

Piezo1 is a mechanically-activated, non-selective cation channel that transduces ECM stiffness into intracellular biochemical signals (Xu et al., 2026; Zhao et al., 2022). In the stiffened fibrotic stroma, elevated lipid bilayer tension opens Piezo1 channels, causing an intracellular Ca^2+^ influx (Jiang et al., 2025; Zhang et al., 2026). This gating activates the Piezo1-YAP/TAZ mechanotransduction axis, often via RCC2-dependent pathways, and promotes Rho/ROCK signaling. This downstream activation induces histone acetylation, which sustains the malignant phenotype even after the initial mechanical stimulus is removed (Boyle et al., 2026; Jiang et al., 2025). Piezo1 exhibits a context-dependent dual role in lung tissues. It functions as a mechanosensory checkpoint in normal lung epithelial cells, where its loss accelerates NSCLC progression. Conversely, its hyperactivation within the TME suppresses local immune surveillance (Huang et al., 2019; Cui et al., 2026).

Piezo1 regulates local mechano-immunological responses. Mechanical stress from the fibrotic ECM activates Piezo1 in infiltrating T lymphocytes, causing actin cytoskeleton depolymerization and T-cell exhaustion. Additionally, this mechanosignaling promotes tolerogenic phenotypes in dendritic cells and polarizes macrophages into an M2-like state (Pang et al., 2024; Wang et al., 2022; Yang et al., 2025). Consequently, pharmacological modulation of Piezo1 is a potential strategy for tumor interception and overcoming resistance to immune checkpoint inhibitors (ICIs). This review specifically focuses on the Piezo1-mediated mechanotransduction networks involved in the transition from IPF to NSCLC. While our primary framework centers on this fibrotic precursor niche, we also explicitly discuss how these mechanobiological principles may apply across broader lung cancer settings characterized by significant desmoplasia. We also discuss emerging mechanotherapeutic strategies and interventions targeting the Piezo1-ECM axis. Modulating this mechanosensory checkpoint may help normalize the fibrotic TME, limit malignant transformation, and restore anti-tumor immunity.

## 2 The fibrosis-to-cancer continuum: ECM stiffening as a pro-tumorigenic driver

The progression from IPF to lung cancer represents a mechanobiological continuum where structural remodeling drives malignant transformation. This pathology involves epidemiological overlap, spatial concordance, and feedback mechanisms centered on ECM remodeling.

### 2.1 Epidemiological and pathological overlap

Lung cancer incidence is significantly elevated in patients with interstitial lung disease (ILD). Population studies indicate that ILD is associated with a 2.16- to 2.91-fold increased hazard ratio (HR) for lung cancer, which persists for over a decade following the initial fibrotic diagnosis (Xu et al., 2025). In a cohort of over 5.4 million participants, preexisting ILD increased lung cancer incidence from 26.2 to 355.4 per 100,000 person-years (Xu et al., 2025). Among IPF patients, the cumulative incidence of lung cancer reaches 26.6% at 10 years (Song et al., 2021; Karampitsakos et al., 2023). Concomitant IPF increases the mortality risk of lung cancer (HR 1.99), reducing 5-year survival rates from 30.1% to 14.5% compared to patients without IPF(Kim et al., 2021; Morales Chacón et al., 2025; Wang et al., 2021). However, treatment with antifibrotic therapies (e.g., nintedanib, pirfenidone) is independently associated with a 64% reduction in lung cancer risk (Kijlertsuphasri et al., 2026).

Spatially, approximately 78% of IPF-associated lung malignancies develop in the peripheral lower lobes, adjacent to or within fibrotic honeycombing lesions (Tzouvelekis et al., 2019; Zhang Y. et al., 2025; Erten et al., 2026). This distribution relates to the mechanobiology of honeycomb cysts. In these regions, condensed fibrous tissue forms a rigid microenvironment (8–25 kPa), and elevated mechanical stretch forces promote abnormal bronchiolar proliferation (Wang and Yang, 2022). Histologically, these tumors primarily present as squamous cell carcinoma (37%–46%) or small cell carcinoma. Additionally, 65% exhibit rare bronchiolar histotypes, such as invasive mucinous adenocarcinoma. This histological pattern suggests that transformed small airways within honeycomb cysts serve as the primary sites of tumor origin (Caliò et al., 2017; Xu et al., 2025).

This continuum is supported by shared genetic and epigenetic factors. Mutations in telomere-related genes (TRGs), specifically rare variants in TERT, RTEL1, and PARN, are enriched in this patient population. These mutations impair telomere length maintenance and bypass the replicative barrier, contributing to genomic instability that facilitates the transition from fibrotic senescence to malignant proliferation (Dressen et al., 2018; Peljto et al., 2023; Borie et al., 2023). Furthermore, the MUC5B promoter polymorphism (rs35705950) serves as a major genetic risk factor. The T risk allele increases IPF susceptibility up to 20-fold and induces ectopic expression of mucin-5B in alveolar type II (AT2) cells. The resulting glycoprotein accumulation induces endoplasmic reticulum (ER) stress, indicated by co-localization with the unfolded protein response mediator XBP1. This chronic ER stress creates a pro-inflammatory distal airspace that promotes carcinogenesis (Ryerson et al., 2025; Seibold et al., 2011; Blumhagen et al., 2025). Epigenetically, DNA hypomethylation and histone modifications drive aberrant Wnt/β-catenin and PI3K/Akt signaling. These pathways promote AT2 cell hyperproliferation and squamous metaplasia, serving as a premalignant transition between fibrosis and carcinoma (Tzouvelekis et al., 2019; Fukuizumi and Seike, 2026).

### 2.2 Mechanical properties and matrix remodeling

**FIGURE 1:**
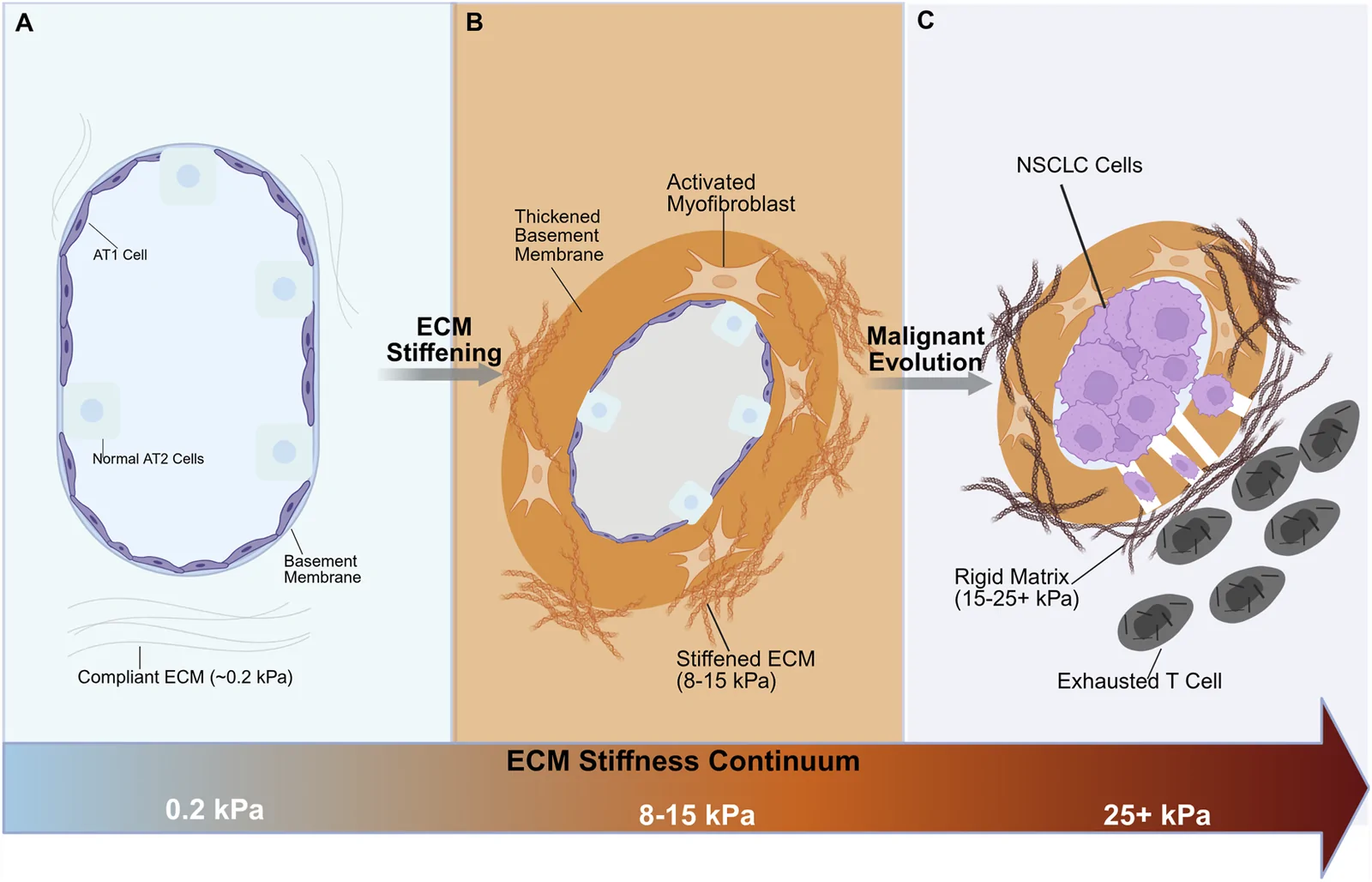
Biomechanical evolution of the lung microenvironment from physiological homeostasis to malignant transformation (Created with BioRender.com). **(A)** Normal Lung Parenchyma. The healthy alveolus maintains physiological homeostasis with a highly compliant extracellular matrix (ECM, ∼0.2 kPa), a thin basement membrane, and normal AT1 and AT2 cells. **(B)** Idiopathic Pulmonary Fibrosis. Pathological mechanical remodeling leads to moderate ECM stiffening (8–15 kPa). Activated myofibroblasts secrete dense, cross-linked collagen bundles, resulting in a thickened basement membrane and structural distortion. **(C)** Malignant Transformation. Extreme matrix rigidity (15–25+ kPa) fundamentally disrupts the lung architecture. NSCLC cells breach the basement membrane and proliferate within a solid tumor nest. This rigid desmoplastic matrix acts as a profound physical barrier, sequestering exhausted T-cell at the tumor periphery and facilitating immune evasion. The bottom gradient illustrates the continuous ECM stiffening that serves as a primary physical driver for the fibrosis-to-cancer continuum.

Biomechanical changes in the fibrotic lung act as physical drivers of carcinogenesis. While clinical and epidemiological data provide strong associative evidence linking matrix rigidity to tumor progression, *in vitro* studies utilizing defined stiffness substrates (e.g., tunable polyacrylamide hydrogels) are crucial for establishing causal mechanisms. For instance, normal lung parenchyma is compliant, exhibiting a physiological stiffness (Young’s modulus) of approximately 0.5–2 kPa (Polio et al., 2018; Guo et al., 2022). Fibrotic remodeling increases local stiffness to 8–25+ kPa. In severe cases, decellularized fibrotic alveolar septa and tunica intima can reach rigidities of 64.8 kPa and 146.6 kPa, respectively (Melo et al., 2014; Mariappan et al., 2014). A stiffness of ∼17 kPa serves as a critical mechanical threshold that induces fibrosis-associated EMT (Brown et al., 2013). The continuous biomechanical and histological evolution from a compliant physiological state to a highly rigid, pro-tumorigenic fibrotic microenvironment is visually summarized in Figure 1.

This stiffening is driven by the accumulation of fibrillar collagens (types I and III) (Guo et al., 2022; Faffe and Zin, 2009). Collagen fibers in the tumorigenic stroma become highly aligned, structurally facilitating cancer cell invasion (Peng et al., 2017). This architectural stabilization is catalyzed by the LOX family, particularly LOXL2. LOX enzymes mediate the oxidative deamination of lysine and hydroxylysine residues, generating reactive allysine intermediates. These intermediates form covalent crosslinks that increase ECM tensile strength and resistance to proteolytic degradation (Boaru et al., 2026; Poller and Wennemers, 2026; Joshi et al., 2026). This creates a positive feedback loop: ZEB1 activation upregulates LOXL2, which crosslinks collagen and stiffens the ECM. The stiffened ECM then amplifies FAK/Src and YAP/TAZ mechanotransduction, which further enhances ZEB1 expression and maintains cells in a pro-invasive mesenchymal state (Peng et al., 2017; Deng et al., 2021).

The translation of these mechanical forces into biochemical signals is mediated by specific mechanotransducers. On stiff matrices (≥15 kPa), YAP and TAZ escape LATS1/2-mediated sequestration and translocate to the nucleus to drive pro-tumorigenic transcription (Liu et al., 2015; Dasgupta and Mccollum, 2019; Panciera et al., 2017). Concurrently, stiff matrices trigger integrin clustering, activating the FAK/Src cascade and the Rho/ROCK pathway. The Rho/ROCK pathway generates actomyosin contractility and induces epigenetic mechanical memory. Specifically, ROCK signaling promotes the acetylation of histone H3 at lysine 9 (H3K9ac) and decreases the trimethylation at lysine 27 (H3K27me3). These epigenetic alterations open chromatin at pro-tumorigenic promoter regions, establishing a malignant and proliferative phenotype that persists after the initial mechanical stress is removed (Young and Reinhart-King, 2023; Boyle et al., 2026).

### 2.3 Fibroblast activation and TGF-β signaling

The fibrotic stroma is heavily populated by CAFs, which represent a highly heterogeneous cellular compartment rather than a singular cell type (Shi et al., 2020; Wong et al., 2022). In NSCLC, this diverse CAF population originates from resident fibroblasts, EMT, endothelial-mesenchymal transition (EndMT), and Smad3-dependent macrophage-mesenchymal transition (Wong et al., 2022; Tang et al., 2022). The transition to an invasive CAF phenotype involves an epigenetic switch initiated by leukemia inhibitory factor (LIF). LIF activates DNMT3b to silence the SHP-1 phosphatase, resulting in constitutive JAK1/STAT3 signaling (Albrengues et al., 2015). Advanced single-cell transcriptomics has profoundly resolved this heterogeneity, categorizing CAFs into distinct functional subsets. These primarily include myofibroblastic CAFs (myCAFs), which represent a specific highly contractile state specialized in excessive ECM deposition and desmoplasia; inflammatory CAFs (iCAFs), which secrete protumorigenic chemokines; and matrix CAFs (THBS2+), which contribute to tumor recurrence (Hirano et al., 2026; Ren et al., 2025). Furthermore, an emerging immunomodulatory subset known as antigen-presenting CAFs (apCAFs), characterized by high MHC-II expression, has been recently identified across various solid tumors including NSCLC (Song et al., 2025). These apCAFs directly interact with T-cell, promoting their activation and enhancing T cell-mediated anti-tumor immunity within the tumor microenvironment.

TGF-β regulates this mechanobiological microenvironment. Through canonical (SMAD2/3) and non-canonical (JNK, p38 MAPK) pathways, TGF-β promotes the differentiation of fibroblasts into the highly contractile myCAF state, upregulates collagens and LOX enzymes, and inhibits matrix degradation via tissue inhibitors of metalloproteinases (TIMPs) (Hashimoto et al., 2001; Enomoto et al., 2023; Shi et al., 2020). Furthermore, ECM stiffness promotes the mechanical activation of latent TGF-β, establishing a self-amplifying fibrogenic loop (Grove et al., 2026).

TGF-β links mechanical remodeling with metabolic and immunological changes. It induces a metabolic shift in CAFs toward aerobic glycolysis by downregulating the pyruvate dehydrogenase complex (PDC) and the mitochondrial enzyme IDH3α. This metabolic alteration stabilizes HIF-1α under normoxic conditions, leading to increased glycolysis and lactate secretion. The secreted lactate is subsequently shuttled to adjacent malignant cells to fuel their mitochondrial metabolism, a process known as the “reverse Warburg effect” (Smith and Hewitson, 2020; Guido et al., 2012). Immunologically, TGF-β represses the proliferation and recruitment of cytotoxic T-cell, contributing to their physical exclusion from the stiffened matrix. Additionally, it promotes the polarization of tumor-associated macrophages (TAMs) toward an immunosuppressive, SPP1+ M2-like phenotype. These TAMs further deposit collagen and impair local immunosurveillance (Henriques et al., 2025; Angioni et al., 2021). Consequently, these TGF-β-mediated processes establish a lactate-rich, immunosuppressive TME that facilitates immune evasion and tumor progression.

## 3 Piezo1 mechanotransduction: the core sensor in the fibrotic niche

Piezo1 mediates the translation of pathological ECM stiffness into intracellular oncogenic signaling. Piezo1 links the physical desmoplasia of the fibrotic lung to the biochemical reprogramming of the TME.

### 3.1 Structure and mechanism of stiffness sensing

Piezo1 is a homotrimeric, propeller-shaped mechanosensitive cation channel (Saotome et al., 2018; Zhao et al., 2018; Jiang et al., 2021). Its gating is primarily governed by the “force-from-lipids' mechanism (Vasileva and Chubinskiy Nadezhdin, 2023; De Vecchis et al., 2021). In the compliant environment of a normal lung, the channel remains in a closed, curved conformation. However, as the fibrotic ECM stiffens to 8–25 kPa, increased lateral membrane tension flattens the channel’s peripheral blades (Yang et al., 2022; Lin et al., 2019). This structural transition initiates a lever-like motion that tilts the pore-lining helices outward, allowing a rapid Ca^2+^ influx into the cytosol (Wang et al., 2018; Liu et al., 2025; De Vecchis et al., 2021). This mechanosensitivity is further amplified by integrin-ECM coupling, which lowers the activation threshold of Piezo1 in the stiffened tumor microenvironment (Lai et al., 2022; Gaub and Müller, 2017).

The mechanosensitivity of Piezo1 to tensile forces is amplified by integrin-ECM coupling. The deposition of fibronectin and crosslinked collagens in the fibrotic stroma sensitizes Piezo1 via focal adhesion complexes containing α5β1 and αvβ3 integrins. These integrins function as molecular tethers that transmit ECM tensile forces to the plasma membrane. This interaction lowers the Piezo1 activation threshold in the stiffened TME (Lai et al., 2022; Gaub and Müller, 2017).

### 3.2 Downstream oncogenic pathways in NSCLC

Following mechanical gating in the fibrotic stroma, Piezo1-mediated Ca^2+^ influx activates intracellular oncogenic signaling cascades. Based on findings from broader solid tumor models, this Ca^2+^ influx is proposed to bypass the canonical Hippo pathway. Instead of relying on upstream LATS1/2 kinases, intracellular Ca^2+^ is suggested to directly activate RCC2 (Regulator of Chromosome Condensation 2), which in turn promotes the nuclear translocation of YAP/TAZ transcriptional co-activators (Jiang et al., 2025; Calderon-Aparicio and Bode, 2021). In the nucleus, YAP/TAZ bind to TEAD transcription factors to upregulate pro-proliferative and pro-invasive genes. Furthermore, the Piezo1-RCC2-YAP axis stimulates autocrine and paracrine TGF-β secretion. This establishes a positive feedback loop that promotes fibrillar collagen deposition and ECM stiffening (Jiang et al., 2025; Duan et al., 2025; Ma et al., 2025). It is important to note that while these Piezo1-mediated signaling cascades are highly relevant to the fibrotic lung TME, many foundational mechanistic insights—such as the RCC2-YAP axis—have been derived from non-lung solid tumor models, including breast, hepatic, and renal carcinomas (Lai et al., 2022; Zhu et al., 2025; Jiang et al., 2025). Although baseline stiffness and stromal composition vary across different tissues, the core principles of Piezo1 mechanotransduction remain widely applicable. Nonetheless, further lung-specific validation is required to definitively confirm the complete execution of these specific cascades within fibrotic NSCLC.

Additionally, sustained Piezo1 activation engages the Ca^2+^/Calpain/YAP proteolytic pathway. In this pathway, calcium-dependent calpain cleavage facilitates YAP nuclear entry. Concurrently, Piezo1 initiates non-canonical EGFR-ERK signaling via Src-p38-dependent serine phosphorylation, which differs from classic ligand-induced tyrosine phosphorylation. These combined pathways promote cell cycle re-entry and EMT (Zhu et al., 2025; Kim et al., 2022; Pardo-Pastor and Rosenblatt, 2023; Kim et al., 2022). In the context of fibrotic ECM stiffening, these cascades primarily converge on the chemical RCC2-YAP mechanotransduction axis to drive malignant progression.

### 3.3 Epigenetic mechanical memory under compressive stress

Beyond matrix stiffening (tensile stress), the physical expansion of rapidly growing tumors within restricted tissue spaces generates substantial compressive forces. Under these conditions, Piezo1 functions as the primary mechanosensor of compression rather than tension. Compressive stress activates Piezo1, initiating an intracellular Ca^2+^ influx that subsequently activates the small GTPase RhoA and its downstream effector, ROCK (Boyle et al., 2026). This Piezo1-mediated Ca^2+^-to-RhoA/ROCK signaling generates the vital intracellular actomyosin contractility necessary to counteract external compression and facilitate cell motility.

**FIGURE 2:**
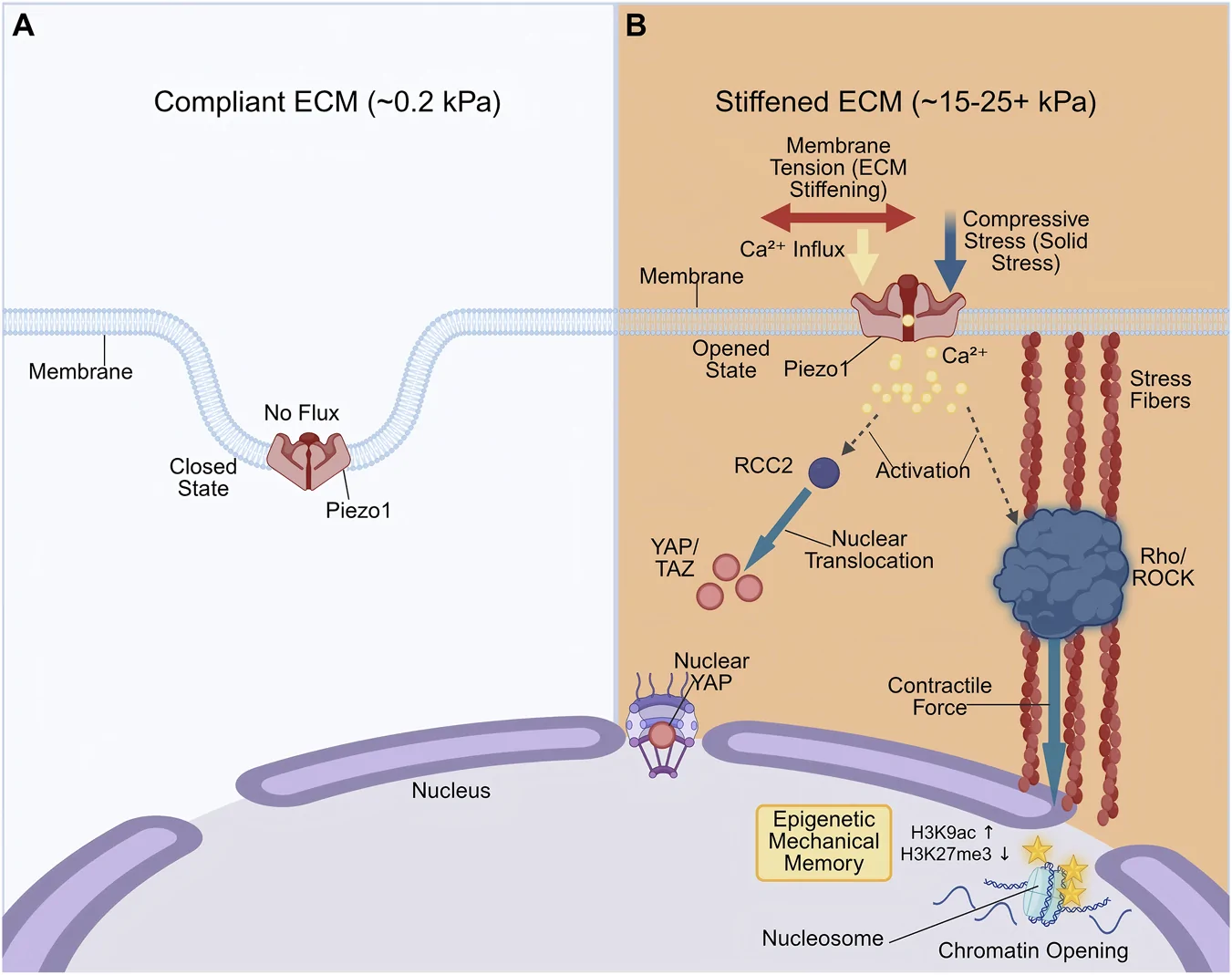
Mechanomolecular mechanisms of Piezo1-mediated gating and bifurcated intracellular signaling cascades under distinct physical stresses (Created with BioRender.com). **(A)** Normal/Soft State. In a compliant ECM (∼0.2 kPa), the lipid bilayer remains unstretched, maintaining Piezo1 in a closed configuration with no ion flux. **(B)** Pathological Mechanical Stress State. The fibrotic tumor microenvironment exerts complex physical forces. Pathological ECM stiffening (∼15–25+ kPa) generates lateral membrane tension, triggering Piezo1-dependent Ca^2+^ influx that primarily activates the chemical RCC2-YAP/TAZ axis for nuclear translocation. Conversely, compressive stress from solid tumor expansion activates Piezo1 to engage the physical Rho/ROCK pathway, generating intense intracellular contractile forces. This physical axis induces an “epigenetic mechanical memory' characterized by enhanced histone acetylation (H3K9ac) and decreased histone methylation (H3K27me3), leading to sustained chromatin opening and pro-malignant transcriptional programs.

Crucially, extrapolations from other invasive carcinoma models indicate that ROCK activation can induce a stable “epigenetic mechanical memory' by altering the chromatin landscape. These specific epigenetic alterations include the enhanced acetylation of histone H3 at lysine 9 (H3K9ac) and the decreased trimethylation of histone H3 at lysine 27 (H3K27me3) (Boyle et al., 2026). These opposing modifications collectively promote a permissive, transcriptionally active chromatin state. This shift sustains the expression of metastasis-promoting genes long after the initial compressive stimulus is removed, establishing a persistent malignant phenotype in invading cancer cells (Boyle et al., 2026; Mertsch and Kr Mer, 2017). The integrated intracellular signaling cascades—specifically bifurcating into the chemical RCC2-YAP axis under ECM tension and the physical Rho/ROCK axis under solid compression—are detailed in Figure 2.

### 3.4 The paradoxical tumor suppressor function in primary epithelium

Despite its pro-tumorigenic actions in the fibrotic stroma, Piezo1 exhibits a context-dependent dual role in NSCLC. In normal, compliant lung epithelium, Piezo1 functions as a physiological tumor suppressor (Xu et al., 2026; Huang et al., 2019). Clinical genomic profiling indicates that the PIEZO1 mRNA transcript is downregulated, or the gene undergoes deep deletion, in primary NSCLC tissues compared to adjacent healthy lung parenchyma. Consequently, elevated Piezo1 expression correlates with increased overall survival and delayed disease progression, particularly in early-stage lung adenocarcinoma (LUAD) (Huang et al., 2019; Wang et al., 2025).

In physiological states, Piezo1 acts as a homeostatic mechanosensor regulating epithelial cell density and tissue architecture. Under sparse cell density, physiological membrane stretch activates Piezo1 to induce compensatory cell division. Conversely, in regions of epithelial crowding, mechanical compression activates Piezo1 to promote live-cell extrusion and apoptosis of metabolically compromised cells, preventing hyperplasia (Gudipaty et al., 2017; Mitchell et al., 2025). Furthermore, Piezo1 mediates cellular quality-control mechanisms. It sensitizes damaged cells to TRAIL-mediated apoptosis via mitochondrial outer membrane permeabilization (MOMP) and mitochondrial depolarization. It also participates in ferroptosis, where lipid peroxidation-induced changes in membrane tension gate Piezo1 to allow cation influx (Kim and Hyun, 2023; Hope et al., 2019; Li et al., 2025).

The genetic or epigenetic abrogation of Piezo1 in primary NSCLC disrupts these mechanical checkpoints. Without functional Piezo1 mechanosensing, transformed epithelial cells evade crowding-induced extrusion, TRAIL-mediated apoptosis, and ferroptosis, resulting in aberrant proliferation (Huang et al., 2019; Hirata et al., 2023). The context-dependent contradictions regarding Piezo1’s function—acting as a tumor suppressor in healthy tissue but transitioning to a pro-tumorigenic driver in the rigid stroma—are explicitly summarized in Table 2. As the primary tumor progresses and the surrounding stroma undergoes fibrotic stiffening, Piezo1 channels on invasive tumor clones are hyperactivated by mechanical stress. In this rigid setting, Piezo1 transitions from a homeostatic mechanosensor to a driver of the RCC2-YAP and Rho/ROCK epigenetic networks (Jiang et al., 2025; Boyle et al., 2026; Silvani et al., 2025). This context-dependent mechanism, regulated by the physical rigidity of the surrounding ECM, emphasizes the need to target the mechanobiological microenvironment with spatial and temporal precision.

## 4 Piezo1-driven immune evasion and microenvironment reprogramming

**FIGURE 3:**
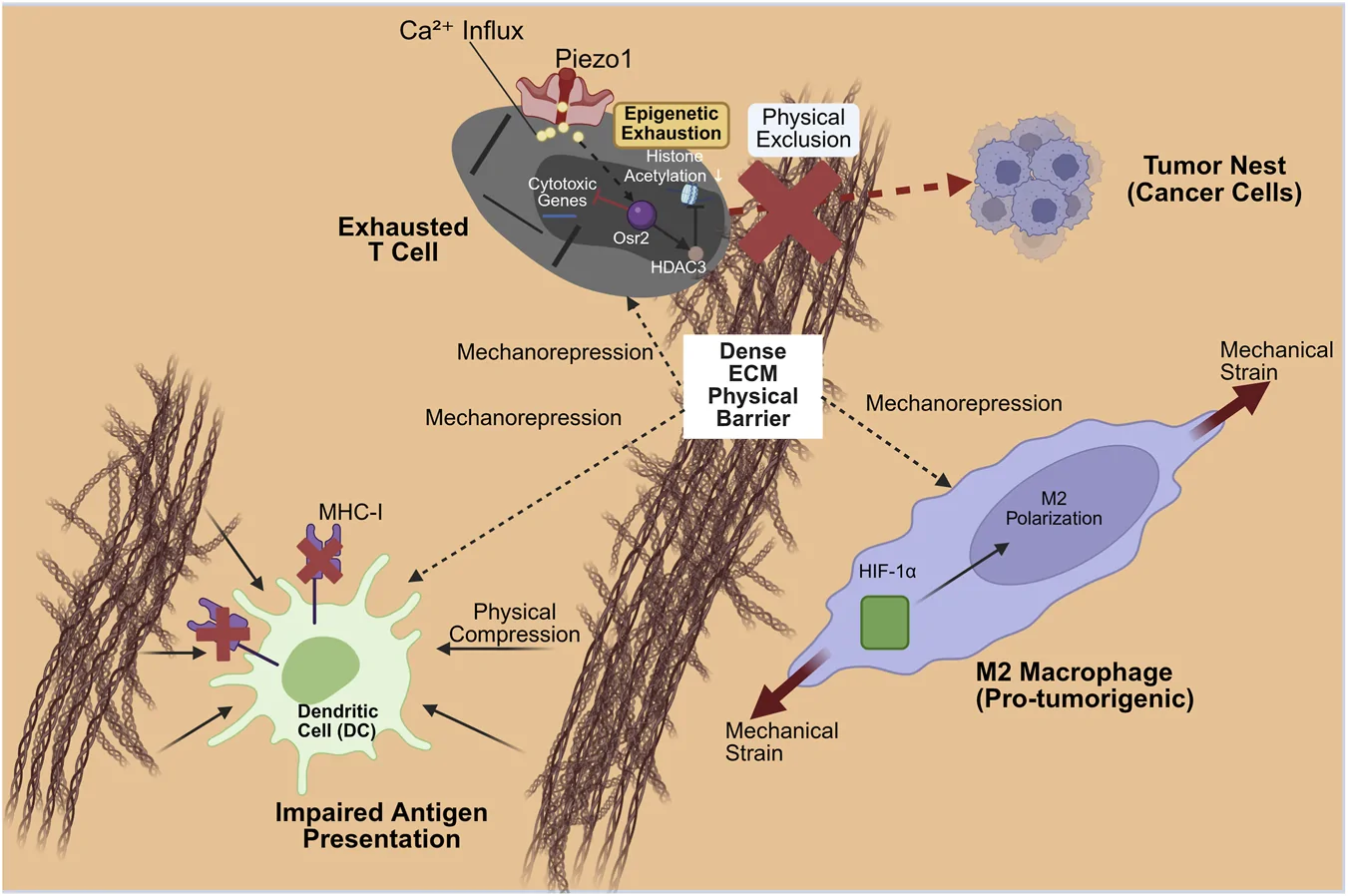
The Piezo1-mediated immune barrier and mechanorepression of anti-tumor immunity in fibrotic microenvironments (Created with BioRender.com). ECM stiffening establishes a dense mechanical barrier that fundamentally remodels the local immune landscape via profound mechanorepression. The hyper-cross-linked collagen network acts as a Dense ECM Physical Barrier, physically excluding immune cells from penetrating the Tumor Nest. Concurrently, this rigid matrix engages Piezo1 on infiltrating immune cells to alter their transcriptional and metabolic states. In T lymphocytes, rapid Piezo1-mediated Ca2+ influx upregulates the Osr2 transcription factor, which recruits HDAC3 to decrease histone acetylation (Epigenetic Exhaustion). This directly represses cytotoxic genes and drives an exhausted phenotype. Simultaneously, sustained mechanical strain stabilizes HIF-1α in tumor-associated macrophages, polarizing them into a pro-tumorigenic M2 state. Furthermore, direct physical compression structurally impedes DCs, impairing MHC-I receptor function and resulting in severely impaired antigen presentation. Collectively, this pathological tension orchestrates a highly immunosuppressive niche by physically excluding T-cell and driving epigenetic exhaustion and tolerogenic polarization.

Traditionally, immune evasion in the TME has been attributed to soluble biochemical factors, such as cytokines and chemokines. However, the physical stiffness of the fibrotic ECM also establishes a “mechano-immunological barrier.' Piezo1 mediates this barrier by regulating the function and metabolic state of adaptive and innate immune cells. This Piezo1-mediated “mechanorepression' is defined here as the impairment of immune cell function induced directly by pathological physical forces. In the following sections, we anchor this conceptual umbrella term to specific cellular data, detailing how matrix stiffness drives epigenetic T-cell exhaustion, macrophage metabolic shifting, and impaired dendritic cell cross-priming (Figure 3).

### 4.1 T-cell exhaustion and matrix stiffness-induced PD-L1

In T lymphocytes, Piezo1 acts as a mechanosensor (Pang et al., 2024; Liu and Ganguly, 2019). During physiological antigen recognition, T-cell receptor (TCR)-pMHC engagement generates mechanical forces at the immunological synapse. This localized mechanical tension activates Piezo1, causing a transient Ca^2+^ influx. This calcium influx activates calpain, which cleaves regulatory proteins to reorganize the cortical actin scaffold. This structural reorganization stabilizes the immunological synapse and facilitates downstream TCR signaling and integrin-dependent migration (Liu et al., 2018; Wu and Ye, 2025; Liu et al., 2024).

In the stiffened fibrotic lung TME, this mechanotransduction pathway promotes T-cell exhaustion (Pang et al., 2024; Zhang et al., 2024). Drawing from recent mechanobiology literature across different tumor contexts, sustained Piezo1 activation by the rigid matrix is hypothesized to engage two immunosuppressive axes in the lung. First, continuous Ca2+ influx may activate the CREB pathway, inducing the expression of the Odd-skipped related 2 (Osr2) transcription factor. Osr2 recruits histone deacetylase 3 (HDAC3) to the promoter regions of effector genes. HDAC3 removes acetyl groups from histones, condensing the chromatin and repressing the transcription of cytotoxic genes (e.g., granzymes, perforin, and interferon-gamma [IFN-γ]). This epigenetic regulation promotes CD8^+^ T-cell exhaustion (Zhang et al., 2024). Second, Piezo1 signaling upregulates the GRHL3-RNF114 pathway. RNF114, an E3 ubiquitin ligase, targets filamentous actin (F-actin) for ubiquitin-proteasome-mediated degradation, leading to cytoskeletal disorganization. This structural impairment reduces the physical traction forces required for T-cell to form functional immunological synapses, impairing their ability to lyse tumor cells (Pang et al., 2024).

Simultaneously, ECM stiffness induces immune checkpoint expression in tumor cells. Matrix rigidity increases intracellular tension in malignant cells via actin stress fiber formation. This tension promotes the nuclear translocation of the transcriptional co-activator YAP (Miyazawa et al., 2018; Lee et al., 2017). In the nucleus, YAP binds to the TEAD enhancer regions of the CD274 (PD-L1) promoter, upregulating PD-L1 expression independently of traditional IFN-γ signaling (Miao et al., 2017). Furthermore, fibrillar collagen in the fibrotic niche binds to leukocyte-associated immunoglobulin-like receptor 1 (LAIR1), an inhibitory receptor expressed on T-cell and myeloid cells. This interaction recruits the SHP-1 phosphatase, which dephosphorylates activating kinases such as Lck and ZAP-70, suppressing TCR signaling (Peng et al., 2020; Pascoal Ramos et al., 2025; Horn et al., 2022). Consequently, mechanical stiffness increases PD-L1 expression on tumor cells while suppressing infiltrating T-cell through cytoskeletal impairment and epigenetic regulation.

### 4.2 Dendritic cell tolerogenesis and macrophage polarization

In addition to adaptive immunity, Piezo1 regulates innate immune cell polarization. Crucially, the function of Piezo1 in innate immunity is highly cell-type specific and state-dependent, sometimes leading to seemingly paradoxical outcomes. In dendritic cells (DCs), Piezo1 influences the balance between tolerogenesis (the induction of immunological tolerance to tumor antigens) and antigen cross-priming (Sánchez-Paulete et al., 2017; Zhu et al., 2026; Wang et al., 2022). For instance, activation of Piezo1 in canonical DCs triggers Ca^2+^-calcineurin-NFAT signaling, which intersects with the SIRT1-hypoxia-inducible factor 1-alpha (HIF-1α) metabolic axis. SIRT1 deacetylates HIF-1α, preventing its degradation and promoting a metabolic shift toward aerobic glycolysis. This metabolic reprogramming alters cytokine production, increasing IL-12 secretion and suppressing TGF-β1. This cytokine profile directs naïve T-cell toward a Th1 anti-tumor phenotype rather than inducing regulatory T-cell (Tregs) (Liu et al., 2015). Conversely, in the CD11 b+ DC subpopulation (specifically cDC2A cells), mechanical stress from the stiff tumor stroma impairs antigen-presenting capability. Genetic deletion or pharmacological blockade of Piezo1 within these CD11 b+ DCs relieves this mechanical suppression. This intervention restores their ability to phagocytose tumor antigens and cross-prime CD8^+^ T-cell, limiting metastatic progression (Bonner et al., 2025).

In TAMs, Piezo1 regulates stiffness-dependent phenotypic polarization (Atcha et al., 2021; Guenther, 2024). Through interactions with integrin CD11 b and downstream CaMKII-HIF-1α pathways, Piezo1 modulates macrophage energy metabolism (Leng et al., 2022; Atcha et al., 2021). In rigid tumors, altered Piezo1 expression promotes an immunosuppressive, oxidative phosphorylation-dependent M2 TAM phenotype, which facilitates tissue repair and angiogenesis. This creates an apparent biological paradox: while chronic, baseline stiffness naturally shifts TAMs toward an M2 state, acute pharmacological hyperactivation of Piezo1 overrides this baseline. Specifically, targeted Piezo1 upregulation or forceful pharmacological activation (e.g., via the small-molecule agonist Yoda1) induces CaMKII-mediated HIF-1α stabilization. This shifts TAMs from oxidative phosphorylation toward glycolytic metabolism, promoting a pro-inflammatory M1 phenotype (Mei et al., 2024; Corcoran and O Neill, 2016; Shin et al., 2025). This demonstrates that therapeutic outcomes depend heavily on the magnitude and method (chronic mechanical vs. acute pharmacological) of Piezo1 activation.

Restoring Piezo1 activity in TAMs using stiffness-tunable soft mesoporous silica nanoparticles increases exosome production. These Piezo1-driven exosomes contain immunostimulatory cargo and specific miRNAs. Upon secretion, these vesicles activate TCR signaling in bystander lymphocytes, enhancing CD8^+^ T-cell infiltration and tumor clearance (Kim et al., 2023; Yu et al., 2023; Peng et al., 2021; Qu and Zhang, 2025; Yang et al., 2025). Furthermore, Piezo1-mediated stiffness sensing is required for macrophage efferocytosis (the phagocytic clearance of apoptotic cells). By clearing dead cells, Piezo1 prevents secondary necrosis and the release of pro-fibrotic damage-associated molecular patterns, facilitating the resolution of pulmonary fibrosis (Wang et al., 2024). Collectively, these mechanisms suggest that pharmacologically targeting Piezo1 is a potential strategy to modulate immunosuppressive myelopoiesis and reprogram the innate immune microenvironment in fibrotic tissues (Aykut et al., 2020).

## 5 Emerging mechanotherapeutic strategies

The recognition that ECM stiffness and Piezo1 activation establish physical and immunological barriers has led to the development of mechano-immunotherapy. This approach aims to normalize the desmoplastic microenvironment and modulate upstream mechanosensors, rather than solely targeting downstream biochemical signaling. Consequently, preclinical models demonstrate that this strategy holds the conceptual promise of converting immunosuppressive “cold' tumors into “hot,' immunotherapy-responsive microenvironments. A comprehensive summary of these emerging mechanotherapeutic agents, detailing their primary targets, distinct mechanisms of stiffness reduction, and synergistic potential with clinical immunotherapies, is provided in Table 1.

**TABLE 1:** Emerging “mechano-immunotherapeutic' agents for lung cancer: targeting the Piezo1-ECM axis to revitalize immunosurveillance.

Target/Pathway	Agent/Class	Primary mechanobiological mechanism	Immunological synergy/Outcome	Clinical/Translational status	Ref.
Piezo1	Yoda1, Yoda2 (KC289), Jedi2 (Small-molecule agonists)	Acts as a “molecular wedge' to lower the mechanical activation threshold; facilitates channel flattening	Triggers YAP-mediated PD-L1 and CXCL10 upregulation; recruits CD8^+^ T-cell; converts “cold' liver metastasis to “hot”	Preclinical	Botello-Smith et al. (2019), Verkest et al. (2025), Parsonage et al. (2023), Zhang T. et al. (2025), Wang et al. (2018)
Piezo1	GsMTx4 (Broad-spectrum gating-modifier peptide)	Partitions into the lipid bilayer; shifts pressure-gating curve to inhibit closed-to-open transition. Broad-spectrum MSIC blocker; partitions into the lipid bilayer; shifts pressure-gating curve to inhibit closed-to-open transition	Prevents GRHL3-RNF114-mediated F-actin degradation; rejuvenates T-cell traction forces and cytotoxicity	Preclinical (Potential for *ex vivo* CAR-T prep)	Pang et al. (2024), Bae et al. (2011), Thien et al. (2024)
Piezo1	Dooku1(Small-molecule antagonist)	Competitively antagonizes Yoda1 binding at the specific allosteric pocket.	Context-dependent modulation (requires caution due to paradoxical agonism in erythrocytes)	Preclinical	Evans et al. (2018), Thien et al. (2024)
LOX Family	β-Aminopropionitrile (β-APN) (Pan-LOX irreversible inhibitor)	Irreversibly blocks active sites of LOX enzymes; prevents new collagen crosslinking	Abrogates TGF-β-induced ECM stiffening; dismantles physical barrier to improve T-cell migration	Preclinical	Tjin et al. (2017), Nicolas-Boluda et al. (2021)
LOXL2/LOXL3	PXS-5153 A (Dual selective small-molecule inhibitor)	Fast-acting inhibition; rapidly dismantles established pathological crosslinks	Severely attenuates matrix rigidity; facilitates microenvironment normalization	Preclinical	Rowbottom et al. (2017), Schilter et al. (2019)
Tyrosine Kinases (PDGFR, FGFR)	Nintedanib, Pirfenidone (Multi-kinase anti-fibrotic agents)	Direct, dose-dependent inhibition of nascent collagen fibril assembly; intercepts TGF-β signaling	Reduces collagen density; alleviates T-cell exclusion barrier	FDA-Approved (IPF)/Clinical Trials	Knüppel et al. (2017), Rangarajan et al. (2016), Meng et al. (2024)
AT1R/TGF-β	Losartan (Angiotensin II receptor blocker)	Suppresses downstream TGF-β signaling; reduces stromal stiffness by ∼28% and increases tumor porosity by ∼45%	Diminishes solid stress and IFP; depletes immunosuppressive CAFs; significantly enhances nanomedicine and T-cell penetration	Clinical Trials (e.g., ARMOR I)	Wang et al. (2026), Dwairy et al. (2025), Hu et al. (2026), Gu et al. (2023)

Abbreviations: AT1R, angiotensin II, type 1 receptor; CAFs, cancer-associated fibroblasts; CXCL10, C-X-C motif chemokine ligand 10; ECM, extracellular matrix; FDA, food and drug administration; FGFR, fibroblast growth factor receptor; IFP, interstitial fluid pressure; IPF, idiopathic pulmonary fibrosis; LOX, lysyl oxidase; PD-L1, programmed death-ligand 1; PDGFR, platelet-derived growth factor receptor; Ref, references; TAMs, tumor-associated macrophages; TGF-β, transforming growth factor-beta.

### 5.1 Piezo1 pharmacological modulators

The pharmacological modulation of Piezo1 provides an experimental strategy for mechanotherapeutic intervention, though it currently remains strictly at the preclinical proof-of-principle stage. High-throughput screening has identified small-molecule agonists, such as Yoda1 and its higher-potency analogue Yoda2 (KC289) (Botello-Smith et al., 2019; Verkest et al., 2025; Parsonage et al., 2023). Additionally, the Jedi1/2 compounds have been identified to activate Piezo1 through a distinct mechanism, utilizing a lever-like transduction pathway by binding to the extracellular side of the peripheral blade domain (Wang et al., 2018). Structurally, Yoda1 binds to a hydrophobic allosteric pocket at the interface of transmembrane helical units 8 and 9, approximately 40 Å from the central pore (Botello-Smith et al., 2019). 3D-MINFLUX nanoscopy indicates that Yoda1 acts as a molecular wedge, facilitating the conformational flattening of the channel blades. This interaction lowers the mechanical activation threshold of Piezo1, inducing Ca^2+^ influx without additional physical stimuli (Botello-Smith et al., 2019; Wijerathne et al., 2022; Lacroix et al., 2018).

Piezo1 antagonism is mediated by agents such as Dooku1, a competitive Yoda1 analogue with a modified pyrazine ring, and GsMTx4, a 34-amino acid amphipathic peptide toxin from Grammostola spatulata venom (Bae et al., 2011; Evans et al., 2018; Thien et al., 2024). Importantly, GsMTx4 is not a specific Piezo1 inhibitor; rather, it is a broad-spectrum blocker of mechanosensitive ion channels. It functions through a lipid bilayer-mediated mechanism instead of direct protein binding (Bae et al., 2011; Cox and Gottlieb, 2019). By partitioning into the outer leaflet of the lipid bilayer, GsMTx4 alters local membrane tension, causing a rightward shift (∼30 mmHg) in the pressure-gating curve. This shift broadly inhibits the closed-to-open transition of the channel without occluding the pore (Bae et al., 2011; Cox and Gottlieb, 2019). The effects of Dooku1 are context-dependent; it acts as an antagonist in endothelial cells but functions as a Piezo1 agonist in red blood cells, indicating that cell-specific membrane lipid compositions influence its activity (Evans et al., 2018; Thien et al., 2024).

These modulators demonstrate potential for combination with ICIs. In metastatic NSCLC, such as liver metastases, the TME lacks the physiological cyclic stretch of the native lung. These metastatic niches exhibit a “cold' phenotype, characterized by diminished PD-L1 expression and reduced CD8^+^ T-cell infiltration (Xiao, 2020; Zhang T. et al., 2025). Administration of a Piezo1 agonist compensates for this mechanical deficit. It mimics mechanical stretch, promoting YAP nuclear translocation, which upregulates tumoral PD-L1 expression and CXCL10 secretion (Zhang T. et al., 2025). The upregulation of PD-L1 provides targets for anti-PD-1/PD-L1 antibodies, while the CXCL10 gradient recruits CD8^+^ T-cell. This combination has demonstrated efficacy in reducing tumor burden in metastatic models (Zhang T. et al., 2025; Knoblauch et al., 2023).

In adoptive T-cell therapies, such as CAR-T cell immunotherapy, *ex vivo* pretreatment of lymphocytes with Piezo1 antagonists (e.g., GsMTx4) offers a potential optimization strategy. Pharmacological blockade of Piezo1 prevents the activation of the GRHL3-RNF114 actin-depolymerizing axis when T-cell enter the stiff tumor stroma. Preserving filamentous actin maintains the cytoskeletal traction forces required for T-cell cytotoxicity and facilitates intratumoral infiltration through the fibrotic barrier (Buglione et al., 2025; Thien et al., 2024; Pang et al., 2024).

Despite the immense potential of Piezo1 pharmacological modulators, their systemic administration presents a critical translational challenge due to the channel’s context-dependent dual role (as outlined in Table 2). For instance, while Piezo1 antagonists effectively halt mechanotransduction in the fibrotic stroma, they risk disabling Piezo1’s essential tumor-suppressive functions (e.g., homeostatic cell extrusion) in adjacent normal epithelium, potentially promoting secondary tumorigenesis (Gudipaty et al., 2017; Mitchell et al., 2025). Conversely, systemic Piezo1 agonists intended to inflame “cold' metastases could inadvertently hyperactivate oncogenic YAP cascades in stiff primary lesions (Jiang et al., 2025). To reconcile this biological complexity, we propose a spatial and contextual therapeutic framework based on the distinct mechanical states of the tumor niche: In the rigid primary fibrotic stroma (8–25+ kPa): Piezo1 is chronically hyperactivated by matrix tension, driving T-cell exhaustion and YAP-mediated tumor progression. In this context, Piezo1 antagonists are logically indicated to halt oncogenic mechanotransduction and alleviate immune suppression. In compliant metastatic niches (e.g., liver metastases): The microenvironment lacks sufficient mechanical stretch, often resulting in an immune “cold' state with low PD-L1 expression. Here, transient use of Piezo1 agonists (e.g., Yoda1) is therapeutically advantageous to mimic mechanical stress, upregulate PD-L1/CXCL10, and actively recruit T-cell for subsequent ICI therapy. Therefore, the decision to inhibit or activate Piezo1 must be strictly guided by the baseline mechanical properties of the specific tumor site.

**TABLE 2:** The context-dependent “double-edged sword' roles of Piezo1 in NSCLC progression.

Tissue context/Microenvironment	Piezo1 status	Primary mechanobiological function	Downstream signaling and cellular effect	Impact on tumorigenesis
Normal Lung Epithelium	Homeostatic expression/Loss of function	Acts as a physiological mechanosensory checkpoint; regulates epithelial cell crowding and homeostatic extrusion	Triggers localized Ca^2+^ signals leading to the extrusion of overcrowded or aberrant cells to maintain epithelial barrier integrity	Tumor Suppressor. (Loss of Piezo1 impairs cell extrusion, leading to epithelial hyperplasia and accelerating malignant progression)
Fibrotic Stroma/TME	Hyperactivated/Upregulated	Transduces pathological mechanical stress (ECM stiffening and solid compression) from the desmoplastic niche	Activates RCC2-YAP (EMT/proliferation), Rho-ROCK (epigenetic mechanical memory), and polarizes innate/adaptive immune cells (tolerogenesis and T-cell exhaustion)	Pro-Tumorigenic and Immunosuppressive. (Hyperactivation promotes metastasis and establishes a formidable “mechano-immunological barrier”)

To circumvent these contradictions, Piezo1 mechanotherapies must be integrated with precision nanodelivery systems. For example, pH-responsive nanocarriers can exploit the characteristic acidity of the TME (pH 6.5–6.8) through mechanisms such as the cleavage of acid-labile bonds, ensuring the on-demand, localized release of modulators while maintaining stability in physiological circulation (Tapponi et al., 2025; Jiang et al., 2025).

Furthermore, active targeting strategies utilizing Fibroblast Activation Protein (FAP)—a transmembrane protease highly expressed on CAFs(Wang et al., 2025)—demonstrate robust preclinical efficacy. FAP-targeted biomimetic nanosystems designed to restore CAFs to a quiescent state have achieved a 78.3% tumor inhibitory rate alongside enhanced cytotoxic T-cell infiltration (Gao et al., 2025). Similarly, FAP-α-responsive co-delivery platforms have been shown to reduce interstitial fluid pressure (IFP) by 4.9-fold, resulting in complete tumor eradication in 66.7% of treated subjects in preclinical models (Xiang et al., 2026). Adapting such dual pH-responsive and CAF-targeted platforms for Piezo1 modulators allows for spatially restricted release exclusively within the acidic, desmoplastic TME (Ma et al., 2024; Ullah et al., 2025; Qu et al., 2025). This targeted approach is essential to maximize microenvironment normalization while sparing adjacent normal lung parenchyma from severe off-target toxicity.

### 5.2 ECM-targeting therapies and microenvironment normalization

In addition to Piezo1 modulation, reducing the mechanical rigidity of the ECM is necessary to alleviate physical T-cell exclusion and improve vascular perfusion. Strategies targeting the LOX family are central to this effort. Non-selective pan-LOX inhibitors (e.g., β-aminopropionitrile, β-APN) and targeted monoclonal antibodies abrogate TGF-β-induced collagen crosslinking and halt EMT in preclinical lung models (Tjin et al., 2017; Nicolas-Boluda et al., 2021; Barry-Hamilton et al., 2010). However, their clinical translation has faced challenges. The clinical failure of the anti-LOXL2 antibody simtuzumab in IPF and liver fibrosis trials prompted mechanistic re-evaluations. Genetic ablation models indicate that LOXL4, rather than LOXL2, primarily mediates pathological collagen crosslinking in pulmonary desmoplasia (Chen et al., 2020; Ma et al., 2023). Consequently, small-molecule inhibitors (e.g., the dual LOXL2/LOXL3 inhibitor PXS-5153 A) are currently being evaluated for their efficacy in disrupting established crosslinks and reducing matrix rigidity (Rowbottom et al., 2017; Schilter et al., 2019).

Beyond targeted LOX inhibition, established anti-fibrotic agents and repurposed cardiovascular drugs exhibit microenvironment-normalizing capabilities. Nintedanib and pirfenidone, standard-of-care agents for IPF, exert anti-fibrotic effects by intercepting TGF-β and PDGF signaling cascades and through direct structural mechanisms. Electron microscopy reveals that both drugs induce dose-dependent inhibition of nascent collagen fibril assembly, resulting in fewer and thinner collagen bundles (Knüppel et al., 2017; Rangarajan et al., 2016; Meng et al., 2024).

**FIGURE 4:**
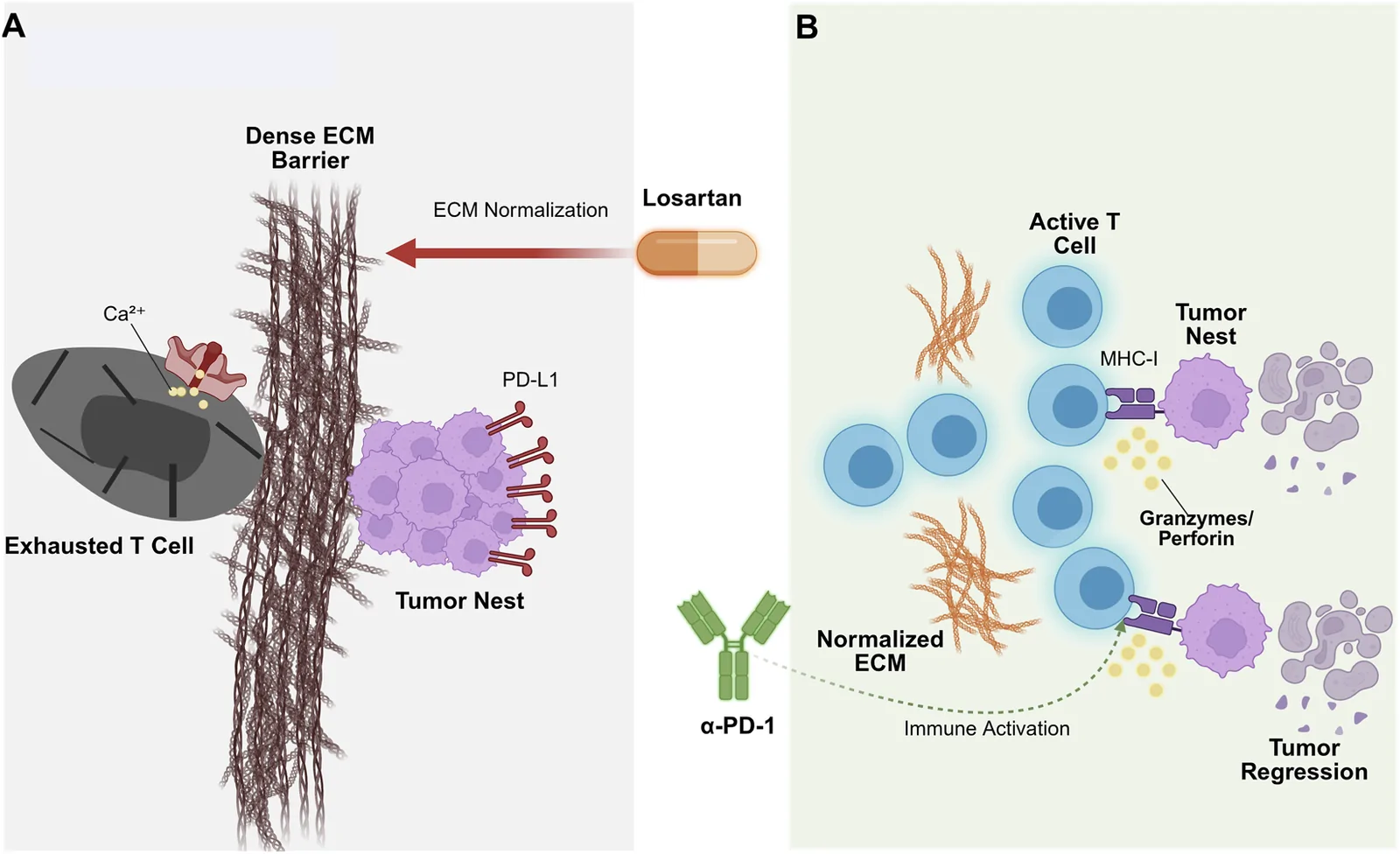
Synergistic therapeutic strategy targeting biomechanical and immunological barriers to reverse immune evasion in NSCLC (Created with BioRender.com). **(A)** Before Treatment. The baseline “cold' tumor microenvironment is characterized by a Dense ECM Barrier that physically excludes immune cells. Mechanical stress drives pathological Ca^2+^ influx in the sequestered, Exhausted T-cell population, while the Tumor Nest exploits high PD-L1 expression to biochemically suppress anti-tumor immunity. **(B)** After Treatment. A dual-therapeutic approach transforms the niche into a highly penetrable “hot' microenvironment. Losartan administration drives ECM Normalization, effectively dismantling the rigid physical shield. Concurrently, α-PD-1 blockade neutralizes biochemical inhibitory signals, driving robust Immune Activation. Within the Normalized ECM, highly Active T-cell successfully infiltrate the tumor core, engage malignant cells via MHC-I recognition, and release cytotoxic Granzymes/Perforin, ultimately culminating in profound Tumor Regression.

The repurposing of losartan, an FDA-approved angiotensin II receptor blocker (ARB), offers immediate translational potential in oncology. Although primarily demonstrated in heavily desmoplastic breast, pancreatic, and hepatocellular carcinoma models—where baseline tissue stiffness may differ from that of the lung—the underlying mechanical principles of AT1R blockade are highly relevant to the fibrotic lung TME. In these models, losartan preconditioning reduces overall stromal stiffness by up to 28%, depletes immunosuppressive CAFs, and increases tumor porosity by approximately 45%. (Wang et al., 2026; Dwairy et al., 2025; Hu et al., 2026; Gu et al., 2023). This mechanical normalization decreases elevated interstitial fluid pressure (IFP) and alleviates compressive solid stress, mitigating the dense collagen barrier. This reduction in physical barriers facilitates the penetration of therapeutic nanomedicines and the infiltration of effector T-cell into the tumor core (Hu et al., 2026; Gu et al., 2023). Clinical trials, such as the ARMOR I trial, utilize ARBs to reshape the TME and resensitize fibrotic NSCLC to chemo-immunotherapy regimens (Gu et al., 2023; Wang et al., 2026). The synergistic therapeutic paradigm of combining ECM-normalizing agents (e.g., Losartan) with immune checkpoint blockade to successfully convert immunosuppressive ‘cold’ tumors into highly active ‘hot’ microenvironments is illustrated in Figure 4.

### 5.3 Integration with existing regimens: Sequencing and toxicity

The clinical translation of mechano-immunotherapeutics necessitates optimizing their integration with standard ICI regimens, specifically regarding administration sequencing and overlapping toxicities. Current preclinical evidence and mathematical modeling strongly support a sequential “prime-then-treat' schedule over concurrent administration. Mathematical models integrating tumor-immune-stromal dynamics demonstrate that stroma normalization provides additive benefits to ICI efficacy when administered as a sequential pre-conditioning phase, while high-dose concurrent strategies can be counterproductive (Mpekris et al., 2020; Swamy, 2022). Preclinical studies suggest that administering ECM-normalizing agents (e.g., LOX inhibitors or losartan) prior to ICI therapy creates a transient “normalization window,' a concept currently being evaluated for broader clinical readiness. During this finite period, interstitial fluid pressure drops, tumor stiffness decreases, and perfusion improves, thereby maximizing the subsequent intratumoral infiltration of cytotoxic T lymphocytes (Nicolas-Boluda et al., 2021; Mirala et al., 2026). For instance, in orthotopic pancreatic cancer models, losartan pre-conditioning effectively opened a compliant state that enabled the deep penetration of subsequent chemo-immunotherapy nanoconjugates (Hu et al., 2026).

Furthermore, the potential for combination toxicity remains a critical clinical hurdle. Systemic mechanical intervention, such as broad ECM degradation, risks impairing physiological tissue homeostasis and wound healing. Although ICI monotherapy generally does not impair surgical wound healing (Kuo et al., 2025), a meta-analysis of 16,976 patients revealed that perioperative ICIs significantly increase grade 3–4 treatment-related adverse events (Fujiwara et al., 2024). When combined with the immune-related adverse events (irAEs) associated with ICIs, particularly checkpoint-induced pneumonitis—which accounts for 15% of fatal ICI toxicities (Schneider et al., 2021)—indiscriminate stromal remodeling could exacerbate alveolar damage and compromise lung structural integrity. Interestingly, recent evidence suggests that specific anti-fibrotic agents, such as nintedanib and pirfenidone, might paradoxically serve as pneumonitis protectants or rescue therapies in patients receiving ICIs (Yamaguchi et al., 2024; Li et al., 2024). Consequently, the successful integration of mechanotherapies with existing regimens relies on utilizing precision nanodelivery systems and establishing optimal sequential dosing intervals to decouple targeted anti-fibrotic efficacy from systemic pulmonary toxicity.

## 6 Conclusions and future perspectives

The Piezo1 ″mechano-immune axis' provides a framework for understanding cancer immunotherapy resistance. The fibrotic TME in NSCLC presents a dual barrier: the physical exclusion of effector T-cell by the dense collagenous matrix, and the stiffness-induced epigenetic exhaustion of T-cell and tolerogenic polarization of innate immune cells via mechanosensor activation. This mechanobiological barrier limits the efficacy of standard ICIs.

Translating these mechanobiological findings into clinical practice presents several challenges. A primary limitation is the ubiquitous physiological expression of the Piezo1 channel. Systemic pharmacological modulation via non-selective agonists or antagonists carries a high risk of off-target toxicities. In the cardiovascular system, Piezo1 is required for endothelial shear stress sensing and vascular tone regulation. Systemic modulation could disrupt this balance, potentially inducing arrhythmias or pulmonary hypertension (Coste and Delmas, 2024; Sun et al., 2025). In the hematologic compartment, Piezo1 regulates erythrocyte volume and platelet activation dynamics. Systemic hyperactivation could trigger mechanothrombosis by pre-activating circulating platelets and leukocytes, whereas systemic inhibition might provoke hemolytic anemia (Bachelot-Loza et al., 2026; Zhu et al., 2022; Zhao et al., 2021).

As discussed earlier, the context-dependent dual role of Piezo1 necessitates precise spatial calibration (Zhao et al., 2021). The integration of the aforementioned precision nanomedicines (e.g., stiffness-tunable or CAF-targeted platforms) is not only essential to resolve this local paradox within the lung but also crucial for preventing systemic catastrophes. By strictly confining therapeutic effects to the immunosuppressive TME, targeted delivery effectively minimizes the severe off-target toxicities in the cardiovascular and hematologic systems (Ma et al., 2024; Ullah et al., 2025; Qu et al., 2025). Additionally, *ex vivo* pretreatment of adoptive cell therapies (such as CAR-T cells) with Piezo1 antagonists prior to patient infusion presents a targeted strategy. This approach preserves T-cell cytoskeletal traction forces and protects against mechanical exhaustion prior to their introduction into the stiffened patient stroma (Pu et al., 2024).

The clinical implementation of mechano-immunotherapy requires the establishment and validation of predictive “mechanomarkers' for patient stratification. Integrating non-invasive, high-resolution imaging techniques, such as Magnetic Resonance Elastography (MRE) or Ultrasound Shear Wave Elastography (SWE), can dynamically map three-dimensional spatial tumor stiffness to provide real-time metrics of mechanical desmoplasia. These physical metrics have the potential to predict ICI responsiveness and complement static PD-L1 immunohistochemical scoring (Streibel et al., 2024; Voutouri et al., 2023; Yuan et al., 2022; Qayyum et al., 2019). At the molecular level, quantifying transcriptomic signatures of ECM remodeling (e.g., the C-ECM gene signature) or evaluating PIEZO1 expression profiles in patient core biopsies can help identify fibrotic tumors likely to benefit from mechanotherapies (Mei et al., 2024; Jensen et al., 2021; Chai et al., 2024). Coupling these diagnostic modalities with CAF-targeted nanomedicines and dual-targeting nanoliposomes is necessary to reprogram the fibrotic niche, reduce the physical barrier, and inhibit tumor-promoting exosome signaling (Freag et al., 2024; Xu et al., 2025).

In conclusion, while the conversion of mechanically “cold' and exclusionary tumors into inflamed, immunotherapy-responsive “hot' microenvironments has been elegantly demonstrated as a proof-of-principle in preclinical models, achieving this clinically remains a formidable challenge. This requires a precision medicine approach that integrates multiple strategies: softening the ECM to enhance vascular perfusion and T-cell infiltration, utilizing targeted Piezo1 modulators to restore innate and adaptive immune cell function, and combining these mechanotherapies with standard ICIs. As precision nanodelivery systems and non-invasive mechanical biomarkers advance, pharmacologically targeting the Piezo1-ECM axis represents a potential therapeutic strategy for patients with refractory, fibrotic lung malignancies.
